# Modifiable early-life risk factors for childhood adiposity and overweight: an analysis of their combined impact and potential for prevention^1^,^2^,^3^,^4^

**DOI:** 10.3945/ajcn.114.094268

**Published:** 2014-12-03

**Authors:** Siân M Robinson, Sarah R Crozier, Nicholas C Harvey, Benjamin D Barton, Catherine M Law, Keith M Godfrey, Cyrus Cooper, Hazel M Inskip

**Affiliations:** 1 From the Medical Research Council (MRC) Lifecourse Epidemiology Unit, University of Southampton, Southampton, United Kingdom (SMR, SRC, NCH, BDB, KMG, CC, and HMI); the National Institute for Health Research (NIHR) Southampton Biomedical Research Centre, University of Southampton and Southampton University Hospitals NHS Trust, Southampton, United Kingdom (SMR, NCH, KMG, and CC); NIHR Musculoskeletal Biomedical Research Unit, University of Oxford, Nuffield Orthopaedic Centre, Oxford, United Kingdom (CC); and UCL Institute of Child Health, London, United Kingdom (CML).

**Keywords:** adiposity, childhood obesity, early life, obesity, lifecourse, prevention

## Abstract

**Background:** Early life may be a “critical period” when appetite and regulation of energy balance are programmed, with lifelong consequences for obesity risk. Insight into the potential impact of modifying early-life risk factors on later obesity can be gained by evaluating their combined effects.

**Objective:** The objective was to examine the relation between the number of early-life risk factors and obesity outcomes among children in a prospective birth cohort (Southampton Women's Survey).

**Design:** Five risk factors were defined: maternal obesity [prepregnant body mass index (BMI; in kg/m^2^) >30], excess gestational weight gain (Institute of Medicine, 2009), smoking during pregnancy, low maternal vitamin D status (<64 nmol/L), and short duration of breastfeeding (none or <1 mo). Obesity outcomes examined when the children were aged 4 and 6 y were BMI, dual-energy X-ray absorptiometry–assessed fat mass, overweight, or obesity (International Obesity Task Force). Data were available for 991 mother-child pairs, with children born between 1998 and 2003.

**Results:** Of the children, 148 (15%) had no early-life risk factors, 330 (33%) had 1, 296 (30%) had 2, 160 (16%) had 3, and 57 (6%) had 4 or 5. At both 4 and 6 y, there were positive graded associations between number of early-life risk factors and each obesity outcome (all *P* < 0.001). After taking account of confounders, the relative risk of being overweight or obese for children who had 4 or 5 risk factors was 3.99 (95% CI: 1.83, 8.67) at 4 y and 4.65 (95% CI: 2.29, 9.43) at 6 y compared with children who had none (both *P* < 0.001).

**Conclusions:** Having a greater number of early-life risk factors was associated with large differences in adiposity and risk of overweight and obesity in later childhood. These findings suggest that early intervention to change these modifiable risk factors could make a significant contribution to the prevention of childhood obesity.

## INTRODUCTION

The rapid rise in prevalence of childhood obesity over recent years has prompted widespread research efforts to identify the factors that explain these secular changes (1, 2). However, although children grow up in more “obesogenic” environments than in the past, not all children become overweight. Understanding how individuals interact with their environment and how these interactions predispose some children to gain excess weight are key issues in considering future preventive strategies (3). There is particular interest in understanding the role of environmental factors in early life, because prenatal and early postnatal life may be “critical periods” when appetite and the long-term regulation of energy balance are permanently programmed—with lifelong consequences for risk of excess weight gain (4, 5).

A number of early-life risk factors have been identified, including maternal obesity, excess gestational weight gain, smoking in pregnancy, and short duration of breastfeeding (3, 5–7). These factors are often socially patterned, and they coexist. For example, excess gestational weight gain and shorter duration of breastfeeding are more common in obese mothers (7–9). The clustering of risk factors has been used as the basis of scoring algorithms, with a view to identifying individual children, for whom targeted preventive interventions may be appropriate. In an analysis of data from Europe and the United States, Morandi et al. (10) showed the best predictors of childhood obesity were parental BMI, birth weight, smoking in pregnancy, number of household members, and maternal occupation, whereas in analyses of data from the Millennium cohort in the United Kingdom, the risk factors used were child's sex, parental BMI, birth weight, smoking in pregnancy, infant weight gain, and breastfeeding status (11). But to understand the potential public health impact, the combined effects of modifiable factors must be evaluated. This approach was first used in Project Viva, in which Gillman and colleagues (12) showed that preschool children, whose mothers had excess gestational weight gain and smoked during pregnancy, who were breastfed for less than 12 mo, and who slept for less than 12 h/d in infancy, had a predicted obesity prevalence (BMI >95th centile) of 29% compared with 6% of children who had none of these risk factors. Importantly, these marked differences have been shown to persist (28% compared with 4%) in later childhood, when the children were aged 7–10 y (13).

BMI is a surrogate marker for adiposity, and to our knowledge, the combined effects of early modifiable risk factors on direct measures of adiposity in childhood have not been evaluated. Using data from a UK birth cohort, we examine the role of 5 factors acting in prenatal and early postnatal life. We chose factors that have been shown to be independent predictors of greater adiposity in children either in this cohort or in other studies and that are potentially modifiable through behavior change interventions: maternal obesity before pregnancy (14, 15), excess gestational weight gain (7, 16), maternal smoking in pregnancy (6), low vitamin D status in pregnancy (17, 18), and short duration of breastfeeding (19–21). We examine their combined effects in relation to 3 outcomes determined at 4 and 6 y of age: BMI, adiposity [dual-energy X-ray absorptiometry (DXA)^5^–assessed fat mass], and overweight or obesity [defined according to the International Obesity Task Force (IOTF) BMI cutoffs (22)].

## SUBJECTS AND METHODS

### The Southampton Women's Survey

The Southampton Women's Survey (SWS) is a prospective birth cohort in which the diet, body composition, physical activity, and social circumstances of a general population sample of nonpregnant women, aged 20–34 y, living in Southampton, were characterized. Detail of the study has been published previously (23). Women were recruited through general practices across the city between April 1998 and December 2002. Each woman was invited to take part by letter, followed by a telephone call, when an interview date was arranged. Of the women, 12,583 agreed, representing 75% of all women contacted. Trained research nurses visited each woman at home and collected information about her health, diet, and lifestyle, as well as taking anthropometric measurements. Women who subsequently became pregnant were followed throughout pregnancy; detailed interviews were conducted at 11 and 34 wk of gestation. The growth and development of the SWS children have been assessed at a number of stages in infancy and childhood, and continued follow-up is ongoing. The SWS was approved by the Southampton and South West Hampshire Local Research Ethics Committee (307/97, 153/99w, 005/03/t, and 06/Q1702/104); written informed consent was obtained from all participants.

### Maternal data

Details of the mothers’ sociodemographic background were obtained during the prepregnant interviews; educational attainment was categorized in 6 groups according to highest academic qualification obtained (increasing from none to university degree or higher). Height was measured with a portable stadiometer (Harpenden; CMS Weighing Equipment Ltd.) to the nearest 0.1 cm with the head in the Frankfort plane. Weight was measured with calibrated electronic scales (Seca) to the nearest 0.1 kg (after removal of shoes and heavy clothing or jewelry). These measurements were used to calculate BMI (in kg/m^2^). Among women who became pregnant, smoking status in pregnancy was ascertained at the 11- and 34-wk interviews. At 34 wk of gestation, a venous blood sample was taken and an aliquot of maternal serum was frozen at −80°C. Serum 25-hydroxyvitamin D concentrations were analyzed by radioimmunoassay (Diasorin). This assay measures both 25-hydroxyvitamin D2 and D3. The assay met the requirements of the UK National Vitamin D External Quality Assurance Scheme; intra-assay and interassay CVs were less than 10%. At 34 wk, the research nurses weighed the women again; pregnancy weight gain from before pregnancy to 34 wk of gestation was defined as excessive according to the Institute of Medicine 2009 recommendations (24), as described previously (weekly gains in second and third trimesters: >0.58 kg/wk in underweight women, >0.50 kg/wk in normal-weight women, >0.33 kg/wk in overweight women, or >0.27 kg/wk in obese women) (7). Gestational age at birth was determined by using a computerized algorithm based on menstrual data or, when these were uncertain (34% of women), with ultrasound assessment of fetal anthropometry in early pregnancy.

### Infancy and childhood data

For infants who were breastfed, the date of the last breastfeed was recorded at 6, 12, or 24 mo and was used to define duration of breastfeeding. When the children reached 4 and 6 y of age, subsets were invited to have an assessment of body composition. Children's height was measured with a Leicester height measurer (Seca), and weight was measured by using calibrated digital scales (Seca). These data were used to calculate BMI at 4 and 6 y. DXA with a Hologic Discovery instrument (Hologic Inc.) was used to assess body composition. Fat mass was derived from the whole-body scan with pediatric software (Hologic Inc.). The total X-ray dose for the whole-body scans was approximately 10.5 microsieverts (pediatric scan mode), equivalent to around 1–2 d of background radiation. All scan results were checked independently by 2 trained operators and agreement reached as to their acceptability; scans showing unacceptable movement artifact were excluded. Physical activity level at 4 y was determined according to the child's average number of hours spent “on the move” and time spent watching television each day, as reported by their parent (25). Diet was assessed when the children were aged 3 and 6 y by using an 80-item food-frequency questionnaire (FFQ) (26, 27). In principal component analyses of the FFQ data, the first component at each age (which explains the greatest variance in the dietary data) described a “healthy” dietary pattern, characterized by frequent consumption of fruit, vegetables, and whole-grain cereals (26). We called this a prudent dietary pattern to be consistent with other studies (28, 29). Prudent diet scores at 3 and 6 y of age were calculated by using the prudent dietary pattern coefficients for every food/group on the FFQ and their reported frequency of consumption at the respective ages. The score describes compliance with the prudent dietary pattern and was used as an indicator of the quality of the children's diets at 3 and 6 y.

### Study population

A total of 1981 women became pregnant and delivered a live-born singleton infant before the end of 2003. Six infants died in the neonatal period, and 2 had major congenital growth abnormalities. Of the 1973 mother-offspring pairs who were available for follow-up, 121 mothers delivered before 37 wk of gestation and were excluded. A further 861 mother-child pairs were not included in the analysis because they did not have complete data either on outcome measurements (*n* = 693 without BMI or fat mass at either 4 or 6 y of age) or on early-life risk factors (*n* = 168). Data for the remaining 991 children are presented in this article.

### Statistical analysis

We selected 5 early-life risk factors, on the basis of previous analyses from the cohort that showed they were independently associated with greater childhood adiposity [excess gestational weight gain (7), low vitamin D status (17), and short duration of breastfeeding (20)] or were factors that have well-established links to obesity in children [maternal obesity (2, 4, 15) and smoking in pregnancy (6)]. They were defined as follows: maternal obesity before pregnancy (BMI >30), excess gestational weight gain [according to Institute of Medicine categorization (7)], smoking in pregnancy according to maternal report, low vitamin D status (<64 nmol/L) as defined previously in this population (17), and short duration of breastfeeding (never or <1 mo). Age at introduction of solid foods was not considered because, consistent with recent systematic reviews (30, 31), we have not found independent associations with adiposity in SWS children (20).

Children's fat mass and BMI data were positively skewed at 4 and 6 y of age and thus were log-transformed and converted to have a mean of 0 and an SD of 1. All outcomes at 4 and 6 y were adjusted for sex and age at measurement; fat mass was also adjusted for height to ensure that any associations were independent of children's stature. Overweight and obesity at 4 and 6 y of age were defined according to the IOTF categorization of BMI (22). For BMI and fat mass outcomes, linear regression models were fitted with the risk factor score as a categorical predictor; zero risk factors were used as the baseline. To assess the effect of the trend in risk factor score, the same models were fitted but with risk factor as a continuous variable. Poisson regression models with robust variance were used to calculate the relative risk of being overweight or obese (defined by using IOTF cutoffs) for each number of factors, compared with the baseline of zero risk factors (32). Additional adjustments were made for potential confounding factors: maternal height, parity, age at child's birth, level of educational attainment, and gestational age of the child at birth. In a final analysis, values were imputed for 861 children with missing data by multiple imputation by chained equations, generating 20 imputed data sets. We then reran the models that examined the associations between number of risk factors and obesity outcomes at 4 and 6 y of age.

Because the risk factors considered could be acting as markers of the nature of the child's postnatal environment, final models further adjusted for childhood level of physical activity (assessed at 4 y) and quality of childhood diet (prudent diet scores determined at 3 y and 6 y). Comparisons between the participants studied and those not included were made by using *t* tests for normally distributed continuous variables, Mann-Whitney rank-sum tests for nonnormally distributed continuous variables, and χ^2^ tests for categorical variables. All statistical analyses were performed with Stata version 13.1 (StataCorp LP).

## RESULTS

The characteristics of the SWS mothers and children studied are shown in **Table 1**. The median ages at the 2 DXA assessments were 4.11 y (IQR: 4.08–4.16 y) and 6.65 y (IQR: 6.47–6.85 y). Compared with the other 861 mothers in the SWS cohort who had term babies born before the end of 2003, those included in the analyses were slightly taller and older; they had higher levels of educational attainment and were more likely to be of white ethnicity, primiparous, and nonsmokers in pregnancy. However, there were no differences in BMI before pregnancy or in their pattern of gestational weight gain. The SWS children who were included in the analyses had comparable BMI at 6 y to the remaining children (Table 1) but were more likely to have been breastfed for longer (*P* < 0.001).

**TABLE 1:** Characteristics of 991 mothers and children studied, compared with the rest of the Southampton Women's Survey cohort, born before the end of 2003

	Mother-child pairs studied		Remaining mother-child pairs		
	*n*	Value	*n*	Value	*P* value^1^
Mother					
Height, cm	991	163.6 ± 6.4^2^	852	162.8 ± 6.5	0.003
BMI, kg/m^2^	991	24.3 (22.0–27.5)^3^	845	24.2 (22.0–28.1)	0.62
Age at child's birth, y	991	30.4 ± 3.8	859	29.7 ± 3.8	<0.001
Serum vitamin D concentration,^4^ nmol/L	991	61.0 (41.7–87.0)	705	57.0 (40.2–82.4)	0.07
Educational attainment; qualifications >A-level,^5^ *n* (%)	988	293 (29.7)	859	228 (26.5)	0.005
Multiparous, *n* (%)	991	524 (52.9)	860	518 (60.2)	0.001
Smoked in pregnancy, *n* (%)	991	140 (14.1)	852	184 (21.6)	<0.001
Pregnancy weight gain,^6^ *n* (%)	991		703		0.68
Inadequate		216 (21.8)		165 (23.5)	
Adequate		301 (30.4)		204 (29.0)	
Excessive		474 (47.8)		334 (47.5)	
White ethnicity, *n* (%)	991	953 (96.2)	861	800 (92.9)	0.002
Child					
Gestation at birth, wk	991	40.2 (39.3–41.0)	859	40.1 (39.1–41.0)	0.29
Duration of breastfeeding, wk	991	13.0 (1.0–30.4)	758	6.0 (0.1–24.7)	<0.001
BMI at 6 y, kg/m^2^	750	15.8 (14.9–16.9)	124	15.5 (14.9–16.5)	0.21
Overweight/obese at 6 y,^7^ *n* (%)	750	122 (16.3)	124	18 (14.5)	0.62

1 *P* values determined according to *t* test for normally distributed continuous variables, Mann-Whitney rank-sum tests for nonnormally distributed continuous variables, and χ^2^ tests for categorical variables.

2 Mean ± SD (all such values).

3 Median; IQR in parentheses (all such values).

4 Concentration determined in late pregnancy.

5 Educational qualification awarded at 18 y of age.

6 Institute of Medicine 2009 categorization (7, 24).

7 International Obesity Task Force categorization (17).

The prevalence of each of the 5 early-life risk factors examined in the analyses is shown in **Table 2**. BMI and fat mass at 4 and 6 y of age are shown in relation to the individual risk factors in **Supplemental Table 1**. When the children were considered in terms of the number of risk factors that they had, 148 (15%) of the children studied had none, 330 (33%) had 1, 296 (30%) had 2, 160 (16%) had 3, 52 (5%) had 4, and 5 (1%) had all 5. The children who had 4 or 5 risk factors were therefore combined into one group for all subsequent analyses.

**TABLE 2:** Definition and prevalence of early-life risk factors

Risk factor	*n*	Prevalence, %
Maternal obesity before pregnancy^1^	135	14
Excessive gestational weight gain^2^	474	48
Smoked in pregnancy	140	14
Low vitamin D status in pregnancy^3^	531	54
Not breastfed or short duration of breastfeeding^4^	355	36

1 BMI >30 kg/m^2^.

2 Institute of Medicine 2009 categorization (7, 24).

3 Serum vitamin D concentration in late pregnancy <64 nmol/L (17).

4 Never breastfed or <1 mo completed breastfeeding.

**Table 3** shows the children's BMI at 4 y and 6 y, according to the number of early-life risk factors. After taking account of a range of potential confounding influences, there was a clear graded increase in BMI at both ages, in association with an increasing number of risk factors. Further adjustment for childhood level of physical activity and prudent diet score made little difference to the patterns of association [adjusted *β*-trend: 0.21 SD (95% CI: 0.14, 0.28) at 4 y; *β*-trend: 0.25 SD (95% CI: 0.17, 0.33) at 6 y; both *P* < 0.001]. The associations between number of early-life risk factors and adiposity in childhood, using DXA-assessed fat mass, were very similar to those observed with BMI (**Table 4**); clear graded increases in adiposity were found with an increasing number of risk factors. Further adjustment for level of physical activity and prudent diet score did not change the associations [adjusted *β*-trend: 0.17 SD (95% CI: 0.10, 0.25) at 4 y; *β*-trend: 0.22 SD (95% CI: 0.14, 0.30) at 6 y, both *P* < 0.001]. To estimate the difference in fat mass between children with 4 or 5 risk factors and those with none, we used the logged data (Table 4) as follows: at 4 y, the fat mass of children who had 4 or 5 risk factors was 0.71 SD higher than those with no risk factors. Since the SD of the log fat mass was 0.24, this equates to an increase of 0.17 (= 0.71 × 0.24) log kg, which is equivalent to a multiplicative change in fat mass of 1.19 [exp(0.17)] (a difference of 19%). Doing the same calculation with the 6-y data, the difference in fat mass amounted to 47%.

**TABLE 3:** BMI in childhood according to number of early-life risk factors

	BMI at 4 y^1^ (SD)			BMI at 6 y^1^ (SD)		
Number of early-life risk factors	*n*	*β* (95% CI)	*P* value^2^	*n*	*β* (95% CI)	*P* value^2^
0	116	0 (—)	—	109	0 (—)	—
1	233	−0.02 (−0.23, 0.20)	0.89	251	0.05 (−0.16, 0.27)	0.63
2	200	0.21 (−0.02, 0.43)	0.07	224	0.28 (0.06, 0.50)	0.01
3	102	0.54 (0.27, 0.81)	<0.001	119	0.55 (0.30, 0.81)	<0.001
4 or 5	37	0.79 (0.42, 1.16)	<0.001	46	1.16 (0.82, 1.50)	<0.001
*β*-trend^3^	688	0.20 (0.13, 0.27)	<0.001	749	0.25 (0.19, 0.32)	<0.001

1 Adjusted for child's sex, gestational age at birth, and age at measurement, as well as maternal height, education, parity, and age at child's birth.

2 *P* values were determined by linear regression models of BMI (*z* score) on risk factor score (zero risk factors as baseline).

3 *P*-trend was determined by linear regression models of BMI (*z* score) on continuous risk factor score.

**TABLE 4:** Fat mass in childhood according to number of early-life risk factors

	Fat mass at 4 y^1^ (SD)			Fat mass at 6 y^1^ (SD)		
Number of early-life risk factors	*n*	*β* (95% CI)	*P* value^2^	*n*	*β* (95% CI)	*P* value^2^
0	92	0 (—)	—	99	0 (—)	—
1	169	−0.08 (−0.30, 0.13)	0.45	217	0.06 (−0.13, 0.24)	0.54
2	141	0.14 (−0.08, 0.37)	0.21	176	0.25 (0.06, 0.44)	0.01
3	75	0.41 (0.14, 0.69)	0.003	99	0.48 (0.26, 0.70)	<0.001
4 or 5	28	0.71 (0.34, 1.08)	<0.001	31	1.14 (0.82, 1.46)	<0.001
*β*-trend^3^	505	0.17 (0.10, 0.24)	<0.001	622	0.22 (0.16, 0.28)	<0.001

1 Adjusted for child's sex, gestational age at birth, age at dual-energy X-ray absorptiometry assessment, and height, as well as maternal height, education, parity, and age at child's birth.

2 *P* values were determined by linear regression models of fat mass (*z* score) on risk factor score (zero risk factors as baseline for categorical analysis).

3 *P*-trend was determined by linear regression models of fat mass (*z* score) on continuous risk factor score.

**FIGURE 1:**
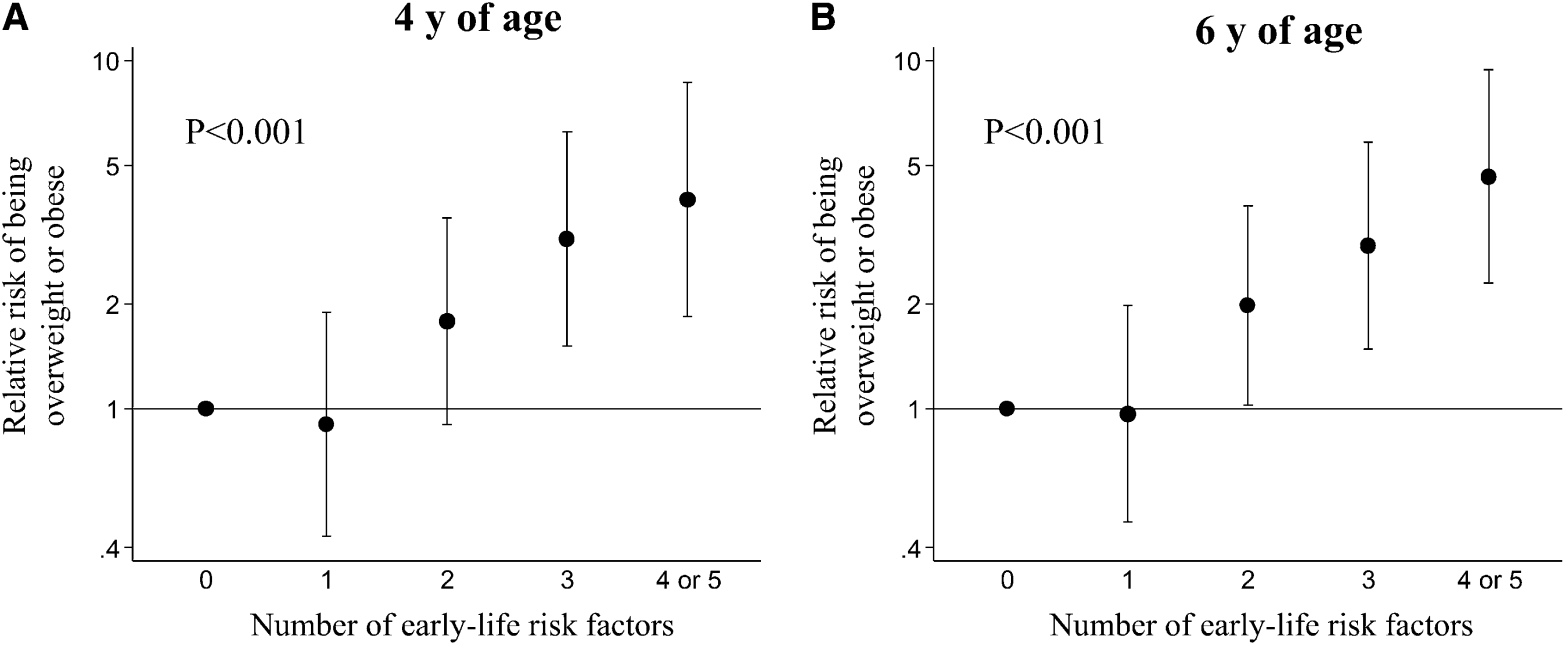
Relative risk (95% CI) of being overweight or obese at 4 and 6 y of age [defined by using IOTF cutoffs (22)], according to number of early-life risk factors (number of children: 116 at 4 y and 109 at 6 y with no risk factors, 233 at 4 y and 251 at 6 y with 1 risk factor, 200 at 4 y and 224 at 6 y with 2 risk factors, 102 at 4 y and 119 at 6 y with 3 risk factors, and 37 at 4 y and 46 at 6 y with 4 or 5 risk factors). Data are adjusted for child's gestational age at birth, as well as maternal height, education, parity, and age at child's birth. *P* values were determined by Poisson regression models with robust variance of IOTF overweight/obese on continuous risk factor score. IOTF, International Obesity Task Force.

According to the IOTF BMI cutoffs, 77 (11.2%) of children were defined as overweight and 19 (2.8%) were obese at 4 y; the figures at 6 y were 97 (12.9%) and 25 (3.3%), respectively. The relative risk of being overweight or obese at 4 or 6 y of age, according to number of early-life risk factors, is shown in **Figure 1**. At both ages studied, there was a graded increase in risk, such that, after taking account of the effects of confounders, the relative risk for children who had 4 or 5 risk factors was 3.99 (95% CI: 1.83, 8.67) at 4 y and 4.65 (95% CI: 2.29, 9.43) at 6 y compared with the children who had none (*P* < 0.001 at both ages). Further adjustment for level of physical activity in childhood and prudent diet score attenuated the associations, but a 4-fold difference in relative risk remained [relative risk for children who had 4 or 5 risk factors: 3.51 (95% CI: 1.55, 7.92) at 4 y and 3.52 (95% CI: 1.56, 7.95) at 6 y].

In our final analyses, we examined the robustness of the associations, first by changing the categorization of 2 risk factors and second by conducting a multiple imputation to address the missing data for 861 children. In the first of these steps, overweight in mothers before pregnancy was defined as a BMI >25 (instead of 30), and short duration of breastfeeding was defined as <6 mo (instead of <1 mo). The models presented in Tables 3 and 4 were rerun first by using each of the new categorizations separately and then both together. For BMI and fat mass at 4 and 6 y, the models were comparable to the original models, although in some cases, the associations were slightly attenuated. For example, in the model that predicted fat mass at 6 y, when both categories were changed (such that 6% of children had no risk factors and 17% had 4 or 5), the *β*-trend was 0.17 SD (95% CI: 0.12, 0.23; *P* < 0.001) compared with 0.22 SD (95% CI: 0.16, 0.28; *P* < 0.001) in the original model, shown in Table 4. However, the effects of changing the definitions of maternal overweight or short breastfeeding duration on the models were small, and the clear positive graded increases in obesity outcomes, in association with increasing number of early-life risk factors, remained; in all models, the positive trends were significant (all *P* < 0.001, data not shown). In the second step, we ran a multiple imputation to impute values for children with missing data and then reran the models that examined the association between number of risk factors and BMI and fat mass at 4 and 6 y. Inclusion of data for these children (*n* = 861) in the models made little difference to the findings, and the pattern of association and size of effects were largely unchanged (data not shown).

## DISCUSSION

We examined 5 early-life risk factors for obesity in childhood (maternal obesity, excess gestational weight gain, smoking in pregnancy, low maternal vitamin D status, and short duration of breastfeeding) that are potentially modifiable through behavior change interventions. By using the categorizations we defined a priori, 15% of the children studied had none of these risk factors, whereas half (52%) had at least 2. We considered BMI, DXA-assessed fat mass, and risk of overweight and obesity as outcomes when the children were aged 4 and 6 y. In each case, we found positive graded associations with the number of early-life risk factors—and the differences were large. For example, there was a 4-fold increase in the risk of being overweight or obese for the children who had 4 or more risk factors, compared with children who had none, and a difference in fat mass between these groups of 19% at 4 y and 47% at 6 y. The associations were still evident after taking account of a range of potential confounding factors, including childhood level of physical activity and diet quality.

To our knowledge, an evaluation of the combined effects of early-life risk factors on adiposity, by using direct measures of fat mass, has not been conducted before. The 4-fold difference in relative risk of being overweight that we observed, when comparing children who had at least 4 early-life risk factors with those who had none, is comparable to data from Project Viva (12). In that study, a 5-fold difference in predicted obesity prevalence in preschool children was described in children whose mothers had excess gestational weight gain and smoked in pregnancy, who were breastfed for <12 mo, and who slept for <12 h/d in infancy (29% compared with 6%). Although some of the risk factors differed between studies (Project Viva included duration of sleep in infancy but did not consider maternal vitamin D status or BMI as individual risk factors), the analyses from both cohorts point to the potential importance of early-life influences for later obesity risk and are suggestive of the public health impact that interventions to change levels of risk factors in early life might achieve.

More recent follow-up of the children in Project Viva has shown that the differences in obesity risk persist, with a 7-fold difference in predicted prevalence between children with all 4 risk factors and those with none, when aged 7–10 y (13). Consistent with this finding, we observed a slightly greater relative risk of overweight or obesity and a larger difference in fat mass at 6 y, suggesting that the differences may widen in later childhood. Such an effect has been described in other studies (33, 34). For example, in an analysis of data from the National Longitudinal Study of Youth, an increasing influence of maternal prepregnancy obesity on child overweight was seen at each follow-up: aged 2–3 y (adjusted OR: 1.37; 95% CI: 1.02, 1.84), aged 4–5 y (OR: 1.69; 95% CI: 1.22, 2.34), and aged 6–7 y (OR: 2.91; 95% CI: 2.09, 4.03) (34). The authors suggested that maternal obesity may affect both the propensity to gain excess weight in childhood and the dynamics of the process. This could be explained by a combination of programmed effects of prenatal exposure to maternal obesity, together with exposure to a more obesogenic postnatal environment. In the present study, we took account of diet quality in childhood and level of physical activity, which slightly attenuated but did not remove the associations. In the continued follow-up of the SWS children, it will be important to address the potential amplification of effects of early-life factors on later childhood obesity, as well as contributions made by factors, such as diet and exercise, to its development in high-risk children.

The SWS provides data from a large contemporary cohort of women and children. A strength of our analyses is that the children and mothers have been characterized in detail, enabling us to take account of effects of a number of potential confounding influences. However, it is a significant limitation that we examined subsets of SWS children at 4 and 6 y of age and that data were not available for all children. The children included in our analyses came from a wide variety of social backgrounds but differed in some characteristics from other SWS children (Table 1). However, maternal BMI and children's BMI at age 6 y did not differ between the groups, and multiple imputation to include children with missing data in the analyses did not change the nature of the associations described. Unless associations differ in the remainder of the cohort, we think it unlikely that selection bias could be the explanation for the findings. We examined both BMI and DXA-assessed fat mass as outcomes in childhood. DXA provides a direct measure of fat mass, and its use is well validated in adults, but there may be challenges with its use in children. To address these, we used specific pediatric software, and movement artifact was modest; the few individuals with excessive movement were excluded from our analyses.

In an observational study, it is not possible to determine whether associations are causal, and there is continuing debate regarding the possibility that early-life factors, such as breastfeeding duration, are acting as markers of the postnatal environment (35, 36). We therefore took account of a range of confounding influences, although there may be other factors that are associated with both early-life risk factors and childhood adiposity that we did not consider. However, although we cannot rule out the possibility of residual confounding, we would not expect it to explain the strong associations we observed.

The size of the differences in adiposity and the risk of being overweight or obese between children who had a number of early-life risk factors and those who had none is an indication of the potential public health significance of early influences on obesity. Body composition “tracks” from early childhood (37, 38), suggesting that these differences will persist into adult life. Although early preventive interventions in childhood are recognized as being important (39), much of the existing evidence of benefit is from school-aged children (40). The SWS data, together with findings from other cohorts (10–13), make a clear case for interventions earlier in the life course, although more evidence is needed. Pregnancy and early infancy have both been considered as times when women will be receptive to health promotion messages (4, 13, 41), and interventions to limit gestational weight gain (42) and promote breastfeeding have been successful (43). But earlier interventions to change levels of risk factors before conception may be a more effective preventive strategy. Two of the risk factors we examined (obesity and smoking) are present before pregnancy. Importantly, they have been shown to coexist with other risk factors in SWS women, such that maternal obesity is associated with a greater risk of excess gestational weight gain and a shorter duration of breastfeeding (7, 44), and maternal smoking is linked to lower vitamin D status in late pregnancy (17). If these are causally related, interventions that address obesity and smoking behaviors before pregnancy will also affect the other risk factors we considered.

Health behaviors are socially patterned and so predict future social inequalities (45). Evidence from the SWS shows there is little preparation for pregnancy in terms of changes in health behaviors, and this is more marked among disadvantaged women (46). Interventions and policies to encourage behavior change need to include recognition of the influence of the wider social and economic environment (47), as well as consideration of how best to support individuals (48). The challenges of changing behavior, to achieve a healthy body weight and stop smoking, are considerable (49, 50). But for women who go on to become pregnant, our findings suggest that the impact of these changes could be significant.

## Supplementary Material

Supplemental data

## Footnotes

5 Abbreviations used: DXA, dual-energy X-ray absorptiometry; FFQ, food-frequency questionnaire; IOTF, International Obesity Task Force; SWS, Southampton Women's Survey.

## References

[1] Lobstein T Baur L Uauy R, for IASO International Obesity Task Force. Obesity in children and young people: a crisis in public health. Obes Rev 2004;5(Suppl 1):4–104. 15096099 10.1111/j.1467-789X.2004.00133.x

[2] Procter KL The aetiology of childhood obesity: a review. Nutr Res Rev 2007;20:29–45. 19079859 10.1017/S0954422407746991

[3] Robinson SM Godfrey KM Feeding practices in pregnancy and infancy: relationship with the development of overweight and obesity in childhood. Int J Obes (Lond) 2008;32:S4–10. 19079279 10.1038/ijo.2008.201

[4] Whitaker RC Dietz WH Role of prenatal environment in the development of obesity. J Pediatr 1998;132:768–76. 9602184 10.1016/s0022-3476(98)70302-6

[5] Reilly JJ Armstrong J Dorosty AR Emmett PM Ness A Rogers I Steer C Sherriff A; Avon Longitudinal Study of Parents. Early life risk factors for obesity in childhood: cohort study. BMJ 2005;330:1357–63. 15908441 10.1136/bmj.38470.670903.E0 PMC558282

[6] Oken E Levitan EB Gillman MW Maternal smoking during pregnancy and child overweight: systematic review and meta-analysis. Int J Obes (Lond) 2008;32:201–10. 18278059 10.1038/sj.ijo.0803760 PMC2586944

[7] Crozier SR Inskip HM Godfrey KM Cooper C Harvey NC Cole ZA Robinson SM; Southampton Women's Survey Study Group. Weight gain in pregnancy and childhood body composition: findings from the Southampton Women's Survey. Am J Clin Nutr 2010;91:1745–51. 20375187 10.3945/ajcn.2009.29128 PMC3091013

[8] Oddy WH Li J Landsborough L Kendall GE Henderson S Downie J The association of maternal overweight and obesity with breastfeeding duration. J Pediatr 2006;149:185–91. 16887431 10.1016/j.jpeds.2006.04.005

[9] Turcksin R Bel S Galjaard S Devlieger R Maternal obesity and breastfeeding intention, initiation, intensity and duration: a systematic review. Matern Child Nutr 2014;10:166–83. 22905677 10.1111/j.1740-8709.2012.00439.x PMC6860286

[10] Morandi A Meyre D Lobbens S Kleinman K Kaakinen M Rifas-Shiman SL Vatin V Gaget S Pouta A Hartikainen AL Estimation of newborn risk for child or adolescent obesity: lessons from longitudinal birth cohorts. PLoS ONE 2012;7:e49919. 23209618 10.1371/journal.pone.0049919 PMC3509134

[11] Weng SF Redsell SA Nathan D Swift JA Yang M Glazebrook C Estimating overweight risk in childhood from predictors during infancy. Pediatrics 2013;132:e414. 23858427 10.1542/peds.2012-3858

[12] Gillman MW Rifas-Shiman SL Kleinman K Oken E Rich-Edwards JW Taveras EM Developmental origins of childhood overweight: potential public health impact. Obesity (Silver Spring) 2008;16:1651–6. 18451768 10.1038/oby.2008.260 PMC2650814

[13] Gillman MW Ludwig DS How early should obesity prevention start? N Engl J Med 2013;369:2173–5. 24224559 10.1056/NEJMp1310577

[14] Gale CR Javaid MK Robinson SM Law CM Godfrey KM Cooper C Maternal size in pregnancy and body composition in children. J Clin Endocrinol Metab 2007;92:3904–11. 17684051 10.1210/jc.2007-0088 PMC2066182

[15] Yu Z Han S Zhu J Sun X Ji C Guo X Pre-pregnancy body mass index in relation to infant birth weight and offspring overweight/obesity: a systematic review and meta-analysis. PLoS ONE 2013;8:e61627. 23613888 10.1371/journal.pone.0061627 PMC3628788

[16] Mamun AA Mannan M Doi SA Gestational weight gain in relation to offspring obesity over the life course: a systematic review and bias-adjusted meta-analysis. Obes Rev 2014;15:338–47. 24321007 10.1111/obr.12132

[17] Crozier SR Harvey N Inskip H Godfrey K Cooper C Robinson S Maternal vitamin D status in pregnancy is associated with adiposity in the offspring: findings from the Southampton Women's Survey. Am J Clin Nutr 2012;96:57–63. 22623747 10.3945/ajcn.112.037473 PMC4632192

[18] Krishnaveni GV Veena SR Winder NR Hill JC Noonan K Boucher BJ Karat SC Fall CH Maternal vitamin D status during pregnancy and body composition and cardiovascular risk markers in Indian children: the Mysore Parthenon Study. Am J Clin Nutr 2011;93:628–35. 21228264 10.3945/ajcn.110.003921 PMC3407368

[19] Arenz S Rückerl R Koletzko B von Kries R Breast-feeding and childhood obesity–a systematic review. Int J Obes Relat Metab Disord 2004;28:1247–56. 15314625 10.1038/sj.ijo.0802758

[20] Robinson SM Marriott LD Crozier SR Harvey N Gale CR Inskip HM Baird J Law CM Godfrey KM Cooper C Variations in infant feeding practice are associated with body composition in childhood: a prospective cohort study. J Clin Endocrinol Metab 2009;94:2799–805. 19435826 10.1210/jc.2009-0030

[21] Owen CG Whincup PH Cook DG Infant and childhood nutrition and disease breast-feeding and cardiovascular risk factors and outcomes in later life: evidence from epidemiological studies. Proc Nutr Soc 2011;70:478–84. 21801475 10.1017/S0029665111000590

[22] Cole TJ Bellizzi MC Flegal KM Dietz WH Establishing a standard definition for child overweight and obesity worldwide: international survey. BMJ 2000;320:1240–3. 10797032 10.1136/bmj.320.7244.1240 PMC27365

[23] Inskip HM Godfrey KM Robinson SM Law CM Barker DJ Cooper C Cohort profile: the Southampton Women's Survey. Int J Epidemiol 2006;35:42–8. 16195252 10.1093/ije/dyi202 PMC4579566

[24] Institute of Medicine. Weight gain during pregnancy: reexamining the guidelines. Washington, DC: National Academies Press; 2009. 20669500

[25] Harvey NC Cole ZA Crozier SR Kim M Ntani G Goodfellow L Robinson SM Inskip HM Godfrey KM Dennison EM Physical activity, calcium intake and childhood bone mineral: a population-based cross-sectional study. Osteoporos Int 2012;23:121–30. 21562877 10.1007/s00198-011-1641-y PMC3685136

[26] Fisk CM Crozier SR Inskip HM Godfrey KM Cooper C Robinson SM Influences on the quality of young children's diets: the importance of maternal food choices. Br J Nutr 2011;105:287–96. 20807465 10.1017/S0007114510003302

[27] Jarman M Fisk C Ntani G Crozier S Godfrey K Inskip H Cooper C Robinson SM; Southampton Women's Survey Study Group. Assessing diets of 3 year old children: evaluation of an FFQ. Public Health Nutr 2014;17:1069–77. 23635946 10.1017/S136898001300102X PMC3743718

[28] Hu FB Rimm E Smith-Warner SA Feskanich D Stampfer MJ Ascherio A Sampson L Willett WC Reproducibility and validity of dietary patterns assessed with a food-frequency questionnaire. Am J Clin Nutr 1999;69:243–9. 9989687 10.1093/ajcn/69.2.243

[29] Slattery ML Boucher KM Caan BJ Potter JD Ma K-N Eating patterns and risk of colon cancer. Am J Epidemiol 1998;148:4–16. 9663397 10.1093/aje/148.1.4-a

[30] Moorcroft KE Marshall JL McCormick FM Association between timing of introducing solid foods and obesity in infancy and childhood: a systematic review. Matern Child Nutr 2011;7:3–26. 21143583 10.1111/j.1740-8709.2010.00284.x PMC6860567

[31] Pearce J Taylor MA Langley-Evans SC Timing of the introduction of complementary feeding and risk of childhood obesity: a systematic review. Int J Obes (Lond) 2013;37:1295–306. 23736360 10.1038/ijo.2013.99

[32] Barros AJD Hirakata VN Alternatives for logistic regression in cross-sectional studies: an empirical comparison of models that directly estimate the prevalence ratio. BMC Med Res Methodol 2003;3:21. 14567763 10.1186/1471-2288-3-21 PMC521200

[33] Hancock KJ Lawrence D Zubrick SR Higher maternal protectiveness is associated with higher odds of child overweight and obesity: a longitudinal Australian study. PLoS ONE 2014;9:e100686. 24955586 10.1371/journal.pone.0100686 PMC4067382

[34] Salsberry PJ Reagan PB Dynamics of early childhood overweight. Pediatrics 2005;116:1329–38. 16322155 10.1542/peds.2004-2583 PMC1479091

[35] Ness AR Griffiths AE Howe LD Leary SD Drawing causal inferences in epidemiologic studies of early life influences. Am J Clin Nutr 2011;94:1959S–63S. 21562085 10.3945/ajcn.110.001461

[36] Kramer MS Oken E Martin RM Infant feeding and adiposity: scientific challenges in life-course epidemiology. Am J Clin Nutr 2014;99:1281–3. 24808493 10.3945/ajcn.114.086181

[37] Deshmukh-Taskar P Nicklas TA Morales M Yang SJ Zakeri I Berenson GS Tracking of overweight status from childhood to young adulthood: the Bogalusa Heart Study. Eur J Clin Nutr 2006;60:48–57. 16132057 10.1038/sj.ejcn.1602266

[38] Freitas D Beunen G Maia J Claessens A Thomis M Marques A Gouveia E Lefevre J Tracking of fatness during childhood, adolescence and young adulthood: a 7-year follow-up study in Madeira Island, Portugal. Ann Hum Biol 2012;39:59–67. 22148930 10.3109/03014460.2011.638322

[39] 1,000 Days. Why 1,000 days [Internet]. Washington, DC: 1,000 Days. 2014 [cited 2014 Aug 13]. Available from: http://www.thousanddays.org/about/.

[40] Waters E de Silva-Sanigorski A Hall BJ Brown T Campbell KJ Gao Y Armstrong R Prosser L Summerbell CD Interventions for preventing obesity in children. Cochrane Database Syst Rev 2011;(12):CD001871. 22161367 10.1002/14651858.CD001871.pub3

[41] Thangaratinam S Rogozinska E Jolly K Glinkowski S Roseboom T Tomlinson JW Kunz R Mol BW Coomarasamy A Khan KS Effects of interventions in pregnancy on maternal weight and obstetric outcomes: meta-analysis of randomised evidence. BMJ 2012;344:e2088. 22596383 10.1136/bmj.e2088 PMC3355191

[42] Tanentsapf I Heitmann BL Adegboye AR Systematic review of clinical trials on dietary interventions to prevent excessive weight gain during pregnancy among normal weight, overweight and obese women. BMC Pregnancy Childbirth 2011;11:81. 22029725 10.1186/1471-2393-11-81 PMC3215955

[43] Renfrew MJ McCormick FM Wade A Quinn B Dowswell T Support for healthy breastfeeding mothers with healthy term babies. Cochrane Database Syst Rev 2012;5:CD001141. 22592675 10.1002/14651858.CD001141.pub4 PMC3966266

[44] Fisk CM Crozier SR Inskip HM Godfrey KM Cooper C Roberts GC Robinson SM; Southampton Women's Survey Study Group. Breastfeeding and reported morbidity during infancy: findings from the Southampton Women's Survey. Matern Child Nutr 2011;7:61–70. 21143586 10.1111/j.1740-8709.2010.00241.x PMC6860776

[45] Lynch JW Kaplan GA Salonen JT Why do poor people behave poorly? Variation in adult health behaviours and psychosocial characteristics by stages of the socioeconomic lifecourse. Soc Sci Med 1997;44:809–19. 9080564 10.1016/s0277-9536(96)00191-8

[46] Inskip HM Crozier SR Godfrey KM Borland SE Cooper C Robinson SM Women's compliance with nutrition and lifestyle recommendations before pregnancy: general population cohort study. BMJ 2009;338:b481. 19213768 10.1136/bmj.b481 PMC2643441

[47] Katikireddi SV Higgins M Smith KE Williams G Health inequalities: the need to move beyond bad behaviours. J Epidemiol Community Health 2013;67:715–6. 23486925 10.1136/jech-2012-202064

[48] National Institute for Health and Care Excellence. 2014. Behaviour change: individual approaches. Public Health Guidance [Internet] [cited 2014 Jun 7];49. Available from: http://www.nice.org.uk/nicemedia/live/14347/66181/66181.pdf.

[49] Hutchesson MJ Hulst J Collins CE Weight management interventions targeting young women: a systematic review. J Acad Nutr Diet 2013;113:795–802. 23473986 10.1016/j.jand.2013.01.015

[50] Amorim Adegboye AR Linne YM Diet or exercise, or both, for weight reduction in women after childbirth. Cochrane Database Syst Rev 2013;7:CD005627. 23881656 10.1002/14651858.CD005627.pub3 PMC9392837

