# Supplementary material for: Modifiable early-life risk factors for childhood adiposity and overweight: an analysis of their combined impact and potential for prevention1
Source: Am J Clin Nutr. 2014 Dec 3;101(2):368–75. doi: 10.3945/ajcn.114.094268 (PMC4307207; doi:10.3945/ajcn.114.094268)
Supplement: Supplemental data [file supp_101_2_368__index.html]

Modifiable early-life risk factors for childhood adiposity and overweight: an analysis of their combined impact and potential for prevention — Supplemental data 

# Modifiable early-life risk factors for childhood adiposity and overweight: an analysis of their combined impact and potential for prevention

## Supplemental data

**Files in this Data Supplement:**

- Supplemental data - Table 1
