# Supplementary material for: Modifiable early-life risk factors for childhood adiposity and overweight: an analysis of their combined impact and potential for prevention1
Source: Am J Clin Nutr. 2014 Dec 3;101(2):368–75. doi: 10.3945/ajcn.114.094268 (PMC4307207; doi:10.3945/ajcn.114.094268)
Supplement: Supplemental data [file 114.094268_ajcn094268SupplementaryData1.docx]

**Online Supplemental Material**

**Table 1. Level of BMI and fat mass in childhood (SD) according to presence of each early life risk factor.**

| **Individual early life risk factors (Yes/No)** | **BMI at 4 years (SD)** | | | |  | **BMI at 6 years (SD)** | | | | |
| --- | --- | --- | --- | --- | --- | --- | --- | --- | --- | --- |
|  |  |  |  |  |  |  |  |  |  |  |
|  | **n** | **β** | **(95% CI)** | **P-value^1^** |  | **n** | **β** | **(95% CI)** | **P-value^2^** |  |
| Maternal obesity before pregnancy^3^ | 688 | 0.44 | (0.22, 0.66) | <0.001 |  | 749 | 0.70 | (0.50, 0.90) | <0.001 |  |
| Excessive gestational weight gain^4^ | 688 | 0.22 | (0.07, 0.37) | 0.005 |  | 749 | 0.32 | (0.18, 0.47) | <0.001 |  |
| Smoked in pregnancy | 688 | 0.45 | (0.22, 0.67) | <0.001 |  | 749 | 0.37 | (0.16, 0.58) | 0.001 |  |
| Low vitamin D status in pregnancy^5^ | 688 | 0.17 | (0.02, 0.32) | 0.02 |  | 749 | 0.14 | (0.00, 0.29) | 0.06 |  |
| Not breastfed or short duration of breastfeeding^6^ | 688 | 0.19 | (0.02, 0.35) | 0.03 |  | 749 | 0.23 | (0.08, 0.39) | 0.004 |  |
| **Individual early life risk factors (Yes/No)** | **Fat mass at 4 years (SD)** | | | |  | **Fat mass at 6 years (SD)** | | | | |
|  |  |  |  |  |  |  |  |  |  |  |
|  | **n** | **β** | **(95% CI)** | **P-value^1^** |  | **n** | **β** | **(95% CI)** | **P-value^2^** |  |
| Maternal obesity before pregnancy^3^ | 505 | 0.36 | (0.13, 0.58) | 0.002 |  | 622 | 0.47 | (0.29, 0.65) | <0.001 |  |
| Excessive gestational weight gain^4^ | 505 | 0.17 | (0.02, 0.33) | 0.03 |  | 622 | 0.26 | (0.14, 0.39) | <0.001 |  |
| Smoked in pregnancy | 505 | 0.33 | (0.09, 0.57) | 0.006 |  | 622 | 0.38 | (0.19, 0.58) | <0.001 |  |
| Low vitamin D status in pregnancy^5^ | 505 | 0.10 | (-0.05, 0.25) | 0.20 |  | 622 | 0.11 | (-0.02, 0.24) | 0.09 |  |
| Not breastfed or short duration of breastfeeding^6^ | 505 | 0.30 | (0.13, 0.47) | <0.001 |  | 622 | 0.26 | (0.12, 0.40) | <0.001 |  |

^1^Adjusted for child’s sex, gestational age at birth, age at measurement; maternal height, education, parity and age at child’s birth; ^2^adjusted for the same factors plus child’s height; ^3^BMI>30 kg/m^2^; ^4^Institute of Medicine 2009 categorisation [7,24]; ^5^Serum vitamin D concentration in late pregnancy <64nmol/l [17]; ^6^Never breastfed or less than 1 month completed breastfeeding
